# The sea lamprey *Petromyzon marinus *genome reveals the early origin of several chemosensory receptor families in the vertebrate lineage

**DOI:** 10.1186/1471-2148-9-180

**Published:** 2009-07-31

**Authors:** Scot Libants, Kevin Carr, Hong Wu, John H Teeter, Yu-Wen Chung-Davidson, Ziping Zhang, Curt Wilkerson, Weiming Li

**Affiliations:** 1 Department of Fisheries and Wildlife, Michigan State University, East Lansing, MI 48824, USA; 2 Research Technology Support Facility, Michigan State University, East Lansing, MI 48824, USA; 3 The Monell Chemical Sense Center, 3500 Market Street, Philadelphia, PA 19104-3308, USA

## Abstract

**Background:**

In gnathostomes, chemosensory receptors (CR) expressed in olfactory epithelia are encoded by evolutionarily dynamic gene families encoding odorant receptors (OR), trace amine-associated receptors (TAAR), V1Rs and V2Rs. A limited number of OR-like sequences have been found in invertebrate chordate genomes. Whether these gene families arose in basal or advanced vertebrates has not been resolved because these families have not been examined systematically in agnathan genomes.

**Results:**

*Petromyzon *is the only extant jawless vertebrate whose genome has been sequenced. Known to be exquisitely sensitive to several classes of odorants, lampreys detect fewer amino acids and steroids than teleosts. This reduced number of detectable odorants is indicative of reduced numbers of CR gene families or a reduced number of genes within CR families, or both, in the sea lamprey. In the lamprey genome we identified a repertoire of 59 intact single-exon CR genes, including 27 OR, 28 TAAR, and four V1R-like genes. These three CR families were expressed in the olfactory organ of both parasitic and adult life stages.

**Conclusion:**

An extensive search in the lamprey genome failed to identify potential orthologs or pseudogenes of the multi-exon V2R family that is greatly expanded in teleost genomes, but did find intact calcium-sensing receptors (CASR) and intact metabotropic glutamate receptors (MGR). We conclude that OR and V1R arose in chordates after the cephalochordate-urochordate split, but before the diversification of jawed and jawless vertebrates. The advent and diversification of V2R genes from glutamate receptor-family G protein-coupled receptors, most likely the CASR, occurred after the agnathan-gnathostome divergence.

## Background

While the general features of the olfactory system are remarkably conserved among vertebrates, the chemosensory receptor gene families expressed there have experienced dramatic diversification [1]. Odor detection and discrimination is accomplished by the interaction of odorous ligands with receptors located on the cilia or microvilli found on the dendritic ends of olfactory receptor neurons (ORNs), and will necessarily depend upon the number of individual receptors and their specificities [2]. In this paper we use the term chemosensory receptor (CR) to refer to all known G protein-coupled receptors that interact with odorant molecules in olfactory epithelia. CR genes have been a subject of much interest since their discovery, because of their persistence in diverse animal taxa, their roles in social and environmental interactions, and their expansion and divergence between vertebrate lineages [3,4]. CR genes exhibit the seven transmembrane domain structure characteristic of the G protein-coupled receptors (GPCR) superfamily and are predominantly expressed in ORNs. Genes encoding large families of candidate CRs have been identified in all vertebrate groups, from lamprey to teleosts, and from amphibians to humans [5,6].

**Figure 1:**
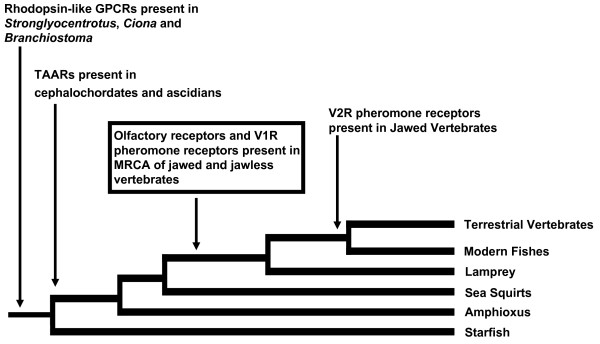
**Proposed evolution of rhodopsin- and glutamate-family chemosensory receptor genes in chordate and vertebrate lineages**. All representative vertebrate genomes listed have been analyzed. Trace amine-associated receptors were present in the common ancestor of chordates. Olfactory- and V1R pheromone receptor genes arose before the divergence of gnathostome and agnathan vertebrate lineages, but are absent from the genomes of those lower chordates described to date. Previous studies show that V2R pheromone receptors are widely diversified in higher vertebrates but have not been identified in the genome or olfactory organ transcriptome of the representative agnathan *Petromyzon*. Chemosensory receptor subfamilies in echinoderms have derived from biogenic amine receptors and have expanded in a lineage-specific manner. Arrows indicate nodes where gene families are first detected.

All studies conducted to date indicate that representatives of OR, TAAR, V1R and V2R gene families were present in the most recent common ancestor of jawed vertebrates [7]. These four families of CR genes display strikingly different evolutionary dynamics and lineage-specific phylogenetic clustering driven by environmental and life history challenges, as well as genome-scale events over the past 450 million years [7-9]. The OR family, for instance, has expanded explosively in the vertebrate lineage and has become the largest gene family described in mammalian genomes [4]. Further, the V1R family has expanded largely in tetrapods and the V2R family has expanded in teleost fishes and amphibians [10]. On the other hand, only a very limited number of potential orthologs of vertebrate OR genes have been identified in the representative genomes of all major phylogenetic groups of invertebrate chordates [11-14]. This striking contrast implicates the significance of CR expansion in the vertebrate radiation, and raises an interesting question on the timing of the origins and initial expansion of all CR gene families (Figure 1). Clearly, genomes representing the jawless stage of vertebrate evolution, the last chordate group whose genomes have yet to be examined systematically for CR families, hold the key information to address this question.

Recently, the sea lamprey genome has been sequenced and made available for examination. Lamprey and hagfishes form a monophyletic group representing the earliest extant primitive vertebrates [15,16]. Extensive electro-olfactogram (EOG) studies indicate that the number of compounds detected by sea lampreys is more restricted than in teleosts [17,18]. The sea lamprey olfactory system is highly sensitive to pheromonal bile acids. However, in direct contrast to teleost olfactory systems, which are highly sensitive to most amino acids, the sea lamprey olfactory system is highly sensitive only to arginine and lysine. Molecular cloning studies in European river lamprey (*Lampetra fluviatilis*) revealed two unrelated families of odorant receptor (OR) genes, one of which resembles typical ORs identified in other vertebrates [19]. The second family shows little sequence identity with any previously described ORs, and is more similar to class A GPCRs for biogenic amines [20]. It is expected that many of these receptors in sea lampreys are expressed in the single, medially positioned main olfactory organ. Posterior to this main organ is an accessory olfactory organ whose epithelial lining may contain cells resembling the ORNs found in the main olfactory epithelium [21]. The olfactory capsule in larvae consists of a small pouch with a region of sensory epithelium containing mature olfactory receptor neurons at the caudal end, and responds to many of the same odorants as in adults [18]. During metamorphosis sea lamprey larvae transform into parasites, possessing eyes, an oral disk and a greatly elaborated olfactory system, feeding by attaching to other fish and consuming blood and body fluids. Parasitic-stage adults cease feeding before migrating into rivers where they become sexually mature, spawn and die. Here we use the term "parasite" to refer to an actively feeding adult removed from free living fish in northern Lakes Huron and Michigan, and the term "adult" to indicate sexually mature lamprey collected from spawning streams. Each of these stages relies upon a functional olfactory system and many of their behaviors are regulated to some extent by odorants [18]. This complex life history offers an opportunity to examine and decipher changes in vertebrate odorant reception at distinct developmental stages.

**Figure 2:**
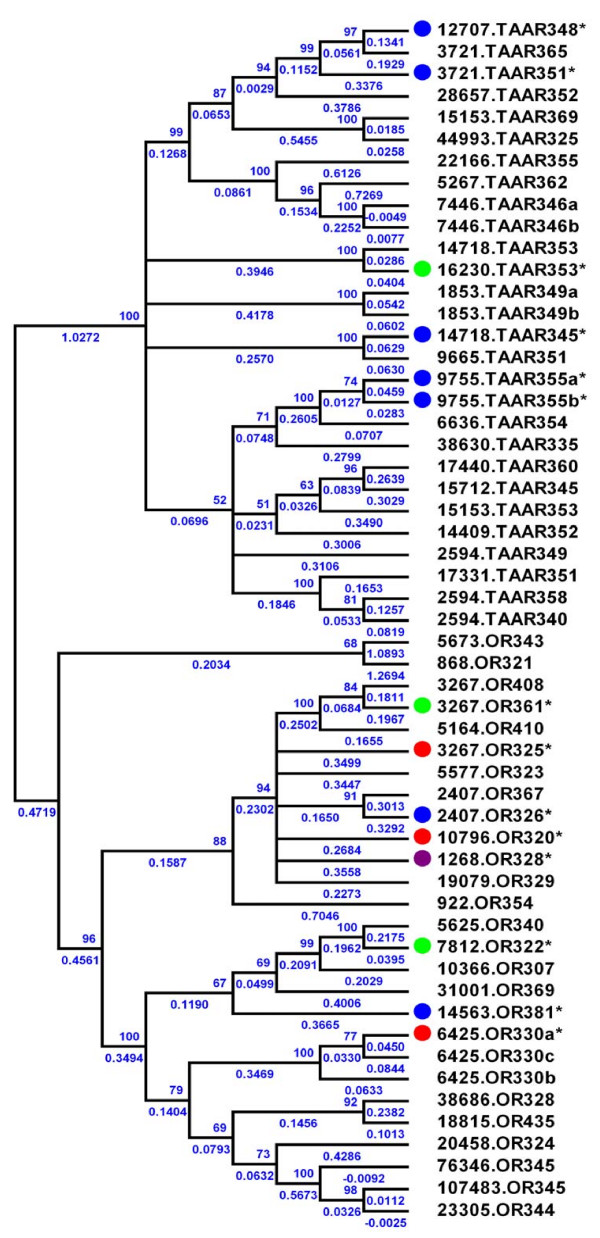
**Phylogenetic analysis of intact single exon OR and TAAR genes in *Petromyzon marinus***. Genes are identified by a supercontig number followed by a gene family designation and predicted amino acid length. The neighbor-joining tree was constructed using CLUSTALW-aligned amino acid sequences and distances were computed using the JTT protein matrix. Confirmed expression is denoted by an asterisk (*), and solid circles represent life stage in which expression was detected by 454 and EST sequencing (red, adult and parasite olfactory organ 454; blue, adult olfactory organ 454; green, parasite olfactory organ 454; and purple, embryo EST). Branch lengths are included and statistical support is given as a percentage of 1000 bootstrap replicates.

Our objective is to determine which CR families were present at the jawless stage of vertebrate evolution and infer the significance of CR genes in the radiation of vertebrates. We present predicted CR genes from the sea lamprey draft genome and describe their expression as determined using Roche 454 GS 20 Life Sciences sequencing technology (Figure 2) and RT-PCR (Table 1), in addition to ESTs from the National Center for Biotechnology Information.

**Table 1:** Sea lamprey olfactory organ CR gene expression from 454, EST and RT-PCR data.

	**olfactory organ 454 transcripts**	**RT-PCR Results**							
**Gene**	**(Adult, Parasite)**	**olfactory **	**brain**	**liver**	**gill**	**kidney**	**gonad**	**RT-primer 5'-3'**	**Tm**

1681.OR230*	**3,4**	**x**					**x**	tcaataatggagtcgcttgc	**60**
								tctcacgttgaaatgccaag	

**2061.V1R320**	**0,2**	**x**	**x**				**x**	gcagtgtcgtggtctctcaa	**56**
								gaatgtagcgcgagttctcc	

**18775.V1R342**	**3,1**	**x**	**x**	**x**	**x**	**x**	**x**	tctgataagcctcgcgttct	**54**
								ctcgggcacatttggtattt	

**6425.OR330**_a_	**1,5**	**x**					**x**	ccgtactgcgactacctcgt	**58**
								ggcactcgtagaggatgagc	

**2407.OR326**	**2,0**	**x**	**x**	**x**			**x**	gctcgaaggatttcaacagg	**57**
								aggtcacggtcctcacaaag	

**3267.OR325**	**7,1**	**x**	**x**				**x**	gtagtggctttgggcatcat	**57**
								cacgaatattgccacgaaaa	

**3721.TAAR351**	**2,0**	**x**					**x**	gtgtggaccttccaccaagt	**60**
								gcagctgcctgaagtagagg	

**9755.TAAR355**_a_	**2,0**	**x**					**x**	gtagccatcgccttcttcag	**60**
								gaggcgtagaaccagcactc	

**16230.TAAR353**	**0,1**	**x**						ctgcgtcgactccttctacc	**52**
								ggaggagttcgtcagcatgt	

**3717.V1R311**	**not detected**	**x**					**x**	tgaagaatggggaactgctc	**54**
								atacaagagcaaccggcatc	

**1548.PRH340**	**not detected**		**x**	**x**			**x**	aacgtgaccaacctgctcat	**54**
								gctgcatgagaaacacgaag	

**11722.V1R311**	**not detected**	**x**						atcaagacgctgctcatcct	**54**
								cccagcagatcacgaatatg	

**5673.OR343**	**not detected**		**x**					atcctcctgtgcaacctgtc	**60**
								ggccggcagatgtagaagta	

**2594.TAAR358**	**not detected**	**x**						tatctcttggctgccgttct	**50**
								gccggagacaaaaacacatt	

**107483.OR345**	**not detected**	**x**						tccacatccagatggtgttc	**60**
								ttggtgttctccaccaggtc	

**17613.OR230**	**not detected,**							atcatggtcacctcctacgc	**60**
	**pseudogene**							ggcaaccagaagatcaggaa	

**9755.TAAR355**_b_	**2,0**							**no RT primers**	

**14718.TAAR353 **	**1,0**							**no RT primers**	

**2008.CASR839**	**1,1**							**no RT primers**	

**10424.MGR935**	**1,1**							**no RT primers**	

**3267.OR361**	**0,1**							**no RT primers**	

1268.OR328**	**0,0**		**NCBI**	**ESTs**	**from**	**embryo**		**EG337313**	
								**EG335699**	

**10796.OR320**	**2,2**							**no RT primers**	

**12707.TAAR348**	**2,0**							**no RT primers**	

**7812.OR322**	**1,0**							**no RT primers**	

**14563.OR381**	**0,1**							**no RT primers**	

**15806.TAP†**	**1,0**							**no RT primers**	

Expression data is shown for a subset of lamprey CR genes, including all OR, TAAR and V1R genes detected by olfactory organ cDNA 454 sequencing. RT-PCR results, primer sequences, annealing temperatures and NCBI EST data are also included. Gonad includes both adult testis and undifferentiated gonad from parasites. The partial gene 1681.OR230 corresponds to three ESTs (EE739806, EE738866 and EB081557) from adult olfactory organ*. Two sequences from the NCBI EST database (EG337313 and EG335699) obtained from sea lamprey embryo map to the gene 1268.OR328**. Expression of a calcium-sensing receptor (2008.CASR839) and a metabotropic glutamate receptor (10424.MGR935) were detected in the olfactory organs of adult sea lamprey. A 540 bp EST represents a cellular nucleic acid binding protein† expressed in both adult olfactory organ and embryo that was not included in the phylogenetic analysis.

## Results

### Chemosensory Receptor Gene Repertoire

An extensive search indicated that the sea lamprey genome contains all three known single-exon CR gene families. TBLASTN searches of the WGS *P. marinus *Draft Genome Assembly 2.0 identified 189 contiguous sequences (contigs) containing potential chemosensory receptor genes (E-values ≤ 1 × 10^-10^). One hundred and eight of these contigs with open reading frames larger than 600 bp were identified by batch BLASTP queries of the NCBI non-redundant protein database as potential CR genes. Potential CRs were considered pseudogenes if they possessed incomplete predicted 7-transmembrane helices, lacked start codons or stop codons, or were highly fragmented. Fifty-two CR pseudogenes were identified, including thirty-five OR pseudogenes and 17 TAAR pseudogenes. Of the one hundred and eight potential CR sequences >600 bp, twelve were identified as histamine-, glycine- and 5-hydoxytryptamine-(5HTR) receptors. Fifty-nine of the one hundred and eight potential CR genes were intact single-exon chemosensory receptors and were included for phylogenetic analysis [see Additional File 1].

Assembly of long scaffolds from the sea lamprey genome has been hindered by the presence of long, repeat-rich regions. Multiple intact CRs occurred on eight contigs, separated by distances ranging from 6.5 kb to 24 kb, with an average distance between intact CR genes of 13.5 kb. Three contigs possessed three CRs (stigs 6425, 3267, 2594), and seven contigs possessed two CRs (stigs 2407, 3721, 7446, 9755, 1853, 14718 15153). The remaining 36 intact CRs occurred singly, and no supercontigs possessed more than three CRs. The calcium-sensing receptors and V1R-like genes described here occurred individually on separate contigs. Intact OR and TAAR genes occurring on the same contig cluster together with high bootstrap support in NJ analysis (Figure 2), and no contigs were found to contain members from more than one CR gene family. NJ analysis of the lamprey calcium-sensing receptor genes and V1R-like genes are presented in Additional Figures 2 and 3, respectively.

Neighbor-joining (NJ), maximum parsimony and maximum likelihood analyses of the intact CR gene nucleotide sequence data set clustered the genes into four well-supported groups, including a group of four V1R-like genes [see Additional Figure 3], a group of twenty-eight TAAR that correspond to the 21 intact TAARs reported by Hashiguchi and Nishida [9], and two well-supported groups (N = 13 and 14, respectively) of ORs (Figure 2). A partial OR cDNA assembled from the 454 data (1681.OR230) mapped to the assembly but was missing its 5' end in both the genome draft and expressed transcripts. This olfactory receptor gene was excluded from the neighbor-joining analysis of intact CRs presented here (Figure 2), but is included in the supplemental material [see Additional File 1].

Two of the predicted intact CR genes from the draft genome (3420.CR393 and 1548.PRH340) were not resolved in the phylogenetic analysis of lamprey OR and TAAR genes and were identified by BLAST homology searches as a putative neuropeptide FF receptor and prolactin releasing hormone receptor, respectively. A single-exon gene (1548.PRH340) in the *Petromyzon *genome demonstrates a high degree of homology with the 2-exon prolactin releasing hormone receptor gene from *Danio rerio *(NP_001034615). While the amino acid sequence of this gene is highly conserved, the exons in the *Danio *ortholog are separated by a 29.4 kb intron containing a predicted *pol *polyprotein (XP_001343585). These putative neuropeptide receptors were also excluded from the phylogenetic analysis in Figure 2.

Neighbor-Joining analysis identifies all lamprey single-exon OR genes as clustering most closely to the θ, η, and κ OR families described by Niimura and Nei [see Additional File 2] [7]. Four intact V1R-like genes (11722.v1r324, 18775.V1R342, 2061.V1R320, 3717.V1R311) cluster in NJ analysis with recently described V1R cDNAs in *Danio *[10,22-24] on the basis of conserved amino acid residues [see Additional Files 3 and 4]. The presence of these V1R-like genes in lamprey indicates that the V1R gene family arose in chordates before the divergence of jawed and jawless fish (Figure 1).

An extensive search of the lamprey draft genome failed to identify likely potential candidates belonging to the multi-exon V2R gene family. PSI-BLAST searches of the *Petromyzon *genome using *Danio *V2R pheromone receptors as queries returned calcium-sensing receptors and metabotropic glutamate receptors, but no candidate V2R genes or V2R pseudogenes. The glutamate and calcium-sensing receptors from lamprey clustered most closely in NJ analysis with V2Rs identified in *Danio *[see Additional File 5]. Using TBLASTN and BLASTP to search the genomes of the echinoderm *Strongylocentrotus purpuratus*, the urochodate *Ciona *and the cephalochordate *Branchiostoma *with a wide variety of V1R and V2R query sequences [see Additional File 2] also failed to identify potential V1R or V2R orthologs in these representative genomes.

### Chemosensory Receptor Gene Expression in Adults and Parasites

Representatives from all maximum likelihood clusters of single-exon CR genes were expressed in the olfactory epithelia of adult and parasitic lamprey (Figure 2). Seventeen of the 59 predicted single-exon CR genes were present in ESTs assembled from 454 reads. Seven of these 17 receptors detected by 454 sequencing were expressed only in the olfactory organs of sexually-mature adults, and four were detected only in the olfactory organ of parasitic-stage adults (Figure 2). Five receptors were expressed in both parasites and adults. Three expressed CRs correspond to the V1R family (2061.V1R320, 3717.V1R311 and 18775.V1R342). No V2R-like genes were detected in the 454 or EST data. Expression of one calcium-sensing receptor and one metabotropic glutamate receptor were detected in the 454 and EST data (Table 1). Publicly available OR-like EST accessions from NCBI correspond to ESTs detected using 454 sequencing of olfactory organ cDNA with the exception of two, EG337313 and EG335699. These accessions represent a single OR gene (1268.OR328) that is expressed in sea lamprey embryos, but was not detected in olfactory tissues of adults and parasites.

454 data from olfactory organ cDNA revealed two potential CR genes that were not predicted from the assembly. A unique CR gene (1681.OR230) that is expressed in both adult and parasite olfactory organ corresponds to three highly similar sequences from the NCBI EST database (EE739806, EE738866 and EB081557). A putative cellular nucleic acid binding protein (15806.TAP) is also expressed in embryo and adult olfactory organ. These partial cDNAs were not included in the phylogenetic analysis. Expression of well-characterized signal transduction cascade components, transient receptor potential cation channel, guanine nucleotide binding protein (G protein), adenylate cyclase, and phospholipase C were also verified in the olfactory organ 454 cDNA data.

**Figure 3:**
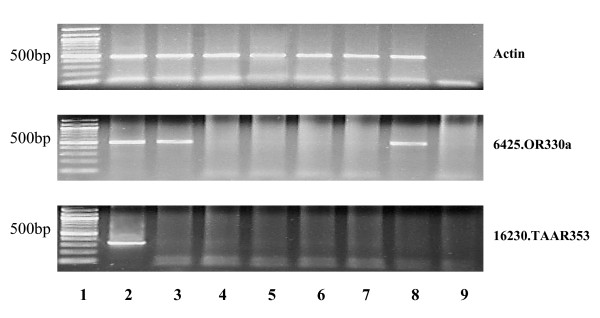
**Chemosensory receptor expression in sea lamprey tissues**. Lane 1: 100 bp DNA ladder; Lane 2: olfactory epithelium; Lane 3: adult sea lamprey testis; Lane 4: brain; Lane 5: liver; Lane 6: gill; Lane 7: kidney; Lane 8: parasite gonad; Lane 9: no template negative control. Reverse transcription PCR (RT-PCR) indicates that representative CR genes are expressed only in olfactory epithelium, testis and in parasite gonad (6425.OR330a, 16230.TAAR353).

**Figure 4:**
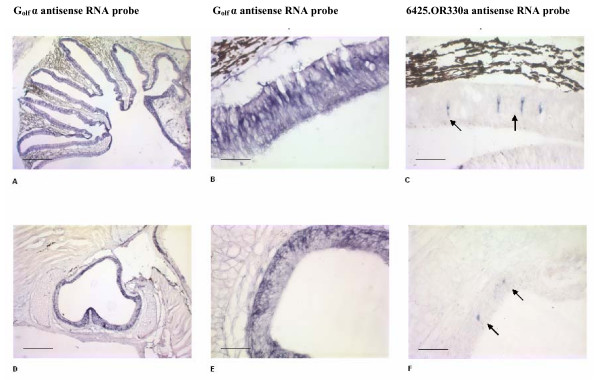
**Chemosensory receptor expression in single olfactory rosettes of adult (A-C) and larval (D-F) sea lamprey**. The left and center panels display olfactory sensory neuron-specific G_olf_α antisense RNA probe. (A) Cross section through the olfactory organ reveals the radial organization of the lamellae in the adult sea lamprey single olfactory rosette. (B) The olfactory epithelium is visible as a band lining the lamellae radiating from the wall of the olfactory chamber. (C) Cells expressing the olfactory receptor (6425.OR330a) in adults are located in the medial region of olfactory epithelium. (D) Note that instead of the convolutions of the adult olfactory lamellae there is a single layer of olfactory epithelium covering the olfactory wall that also expresses G_olf_α in the larval stage (E). (F) The olfactory sensory neurons expressing the olfactory receptor in larval sea lamprey are indicated by arrows. Scale bar: (A, B, D, E) 500 μm; (C, F) 50 μm.

RT-PCR confirmed tissue-specific expression of 6425.OR330a and 16230.TAAR353, representatives of two distinct CR families, the ORs and TAARs, respectively (Figure 3). Expression of 6425.OR330a was restricted to the olfactory epithelia, testis and undifferentiated parasite gonads. 16230.TAAR353 was expressed only in the olfactory epithelium. Expression of 6425.OR330a and 16230.TAAR353 were not detectable in brain, liver, gill, or kidney by RT-PCR. In situ hybridization (ISH) of adult and larval olfactory epithelia found 6425.OR330a to be expressed in the ORN (Figure 4). The olfactory epithelium is visible as a band lining the lamellae radiating from the wall of the olfactory chamber. The columnar cell body of ORN expressing the CR genes in adults are located in the middle layer of the olfactory epithelia (Figure 4C, F). Within this layer, the positive ORNs were randomly distributed within the olfactory epithelium. Individual CR probes annealed with 0.8%–2% of the ORNs in lamprey during larval, parasitic and adult life stages. Olfactory neuron-specific G_olf _α antisense RNA probes also hybridized to this same region. Note that instead of the convolutions of the adult olfactory lamellae, there is a single layer of olfactory epithelium covering the olfactory wall in the larval lamprey that also expresses G_olf _α and 6425.OR330a (Figure 4). Further, ISH indicated that only 6425.OR330a and not 16230.TAAR353 was expressed in the interstitial cells of adult sea lamprey testis (data not shown).

Interestingly, three intact single-exon genes that were poorly resolved between the OR and the V1R-like receptors in our phylogenetic analyses (1548.PRH340, 3420.CR393, and 5673.OR343) were shown to be expressed in the brains of both adult and parasitic lamprey by RT-PCR. Transcripts from the predicted prolactin releasing peptide receptor (1548.PRH340), neuropeptide FF receptor-like gene (3420.CR393) and a novel OR (5673.OR343) were detected by RT-PCR in brain but not olfactory organ, testis or liver from adults and parasites. RT-PCR results and conditions are summarized in Table 1.

## Discussion

The purpose of this study was to identify the repertoire of chemosensory receptors for olfaction in the sea lamprey draft genome and infer the evolution of those gene families in the chordate lineage. All known chemosensory receptors are 7-transmembrane G-protein coupled receptors, but vary considerably in overall sequence and exon structure, consistent with an ability to recognize diverse ligands [7]. This study identifies 59 intact single-exon chemosensory receptor genes in the sea lamprey genome that belong to three CR families: V1R-like, OR and TAAR families. These genes share many characteristics of gnathostome single-exon CR genes. Sea lamprey CR genes identified in this study are similar in structure and size to those described in higher vertebrates [3,4,8]. We found several contigs that each contain highly homologous members of the same CR families, which is consistent with findings in gnathostome genomes where CR genes often cluster in specific chromosomal regions [8]. Furthermore, of the 59 intact CR genes from our *Petromyzon marinus *genome Draft Assembly, we have identified a set of seventeen in the cDNA from olfactory epithelia of mature and parasitic-stage adult sea lamprey using 454 sequencing. RT-PCR and in situ hybridization confirmed that expression of representative OR and TAAR are largely limited to odorant receptor neurons and testis, which is consistent with expression of single-exon CR genes in higher vertebrates [1-3]. The observed life history-specific expression of CR genes may be adaptive for different olfactory-mediated behaviours demonstrated in the sea lamprey, as parasites display search behaviours for detection of prey odor and adults respond specifically to migratory and mating pheromones [25]. It is likely that the majority of the 59 predicted intact genes described in this study are functional CR genes for olfaction in the sea lamprey.

Our data indicate that all three families of single exon CR genes were present in the most recent common ancestor of jawless and jawed vertebrates (Figure 1). Both the OR and TAAR genes are numerous and diverse in the genomes of sea lamprey and higher vertebrates [7,9], supporting an expansion prior to the most recent common ancestor of jawed and jawless vertebrates. Our search for predicted genes from the draft genomes of *Stronglyocentrotus *, *Ciona *and *Branchiostoma * using lamprey gene queries and subsequent phylogenetic analyses suggest that OR, TAAR and V1R gene families had not expanded before the rise of vertebrates, and that OR and V1R receptor families are vertebrate-specific, with TAAR specific to chordates. This is consistent with findings from the genomes of the cephalochordate *Branchiostoma *[11] and the ascidian *Ciona *[14], both possessing trace amine receptors (11 and 14 TAAR, respectively), but neither of which have been shown to possess V1R pheromone or olfactory receptors. A recent report has inferred a much more ancient origin for ORs, placing them in the common ancestor of vertebrates and cephalochordates, while placing the origin of TAARs in the common ancestor of vertebrates [26]. These results are not in agreement with the BLAST results presented here, nor with the available genome-scale analyses of cephalochordates and urochordates [11,14]. A large-scale phylogenetic analysis of available ORs, TAARs and biogenic amine receptors would address this discrepancy. However, given the expanse of time involved in the evolution of these ancestral molecules and the striking variation observed within OR and TAAR gene families, resolution of these discrepancies may require more data than BLAST searches, alignments and phylogenetic inference can provide. Specific ligand-receptor interactions and structural analysis of ligand-binding domains in these receptors should provide critical data regarding homology and convergence in the chemosensory receptor repertoires of deuterostomes.

Analysis of the genome of *Strongylocentrotus purpuratus *has offered strong evidence of an elaborate chemosensory system in echinoderms [27]. Sea urchin rhodopsin-type GPCR repertoires indicate that these large chemosensory receptor subfamilies expanded in echinoderms in a lineage-specific manner and were derived from biogenic amine receptors [28-30]. Using lamprey TAAR and OR to query the GLEAN predicted peptides in the *Strongylocentrotus *genome produced a non-redundant list of 63 serotonin-, octopamine-, histamine and adreno-receptors. Thirteen of these "best hit" receptors that were identified as potential TAARs were included in our phylogenetic analysis. These formed a well-supported clade distinct from single-exon vertebrate chemosensory genes [see Additional File 2]. These results support the hypothesis that chemosensory trace-amine receptors evolved independently at least twice in the deuterostome lineage.

The 28 intact lamprey TAAR genes identified in our study cluster into a well resolved group, in agreement with the 21 TAAR genes identified from a previous sea lamprey draft assembly that form a monophyletic clade [9]. Other than in a few teleost groups, the number of TAAR genes did not expand dramatically in tetrapods [9]. In contrast, OR gene repertoires in both teleosts and tetrapods experienced dynamic expansion before and after these two vertebrate groups diverged. The sea lamprey ORs cluster most closely to the intact η, κ, and θ OR subfamilies described by Niimura and Nei [7]. These OR subfamilies are represented but not expanded in either teleosts or tetrapods [7].

It is interesting that V1R-like genes, a remarkably diverse CR family in tetrapods [10], did not expand extensively in either the sea lamprey or teleost genomes. Sea lamprey V1R-like genes cluster in NJ analysis with expressed V1R genes described in *Danio *[23] [see Additional File 3]. The presence of V1R-like genes in lamprey and our phylogenetic analysis indicate that this family arose early in the vertebrate lineage, before the agnathan-gnathostome divergence (Figure 1), but did not expand dramatically until vertebrates began exploiting terrestrial habitats [10]. Further, the V1R family likely arose after the separation of cephalochordate and urochordate lineages which do not possess identifiable V1R-like sequences in their current draft genomes. The early divergence of V1R in the vertebrate lineage is further demonstrated by the phylogenetic signal observed between the lamprey, teleost and mammalian V1Rs [see Additional File 3] and the need to use an appropriate protein substitution matrix to detect similar homologies across greater than 300 million years of evolutionary time [31]. During the successful diversification in the teleost lineage, the V1R family did not undergo the dramatic expansions that are observed in the genomes of terrestrial vertebrates [10], nor did V1R expand during additional rounds of genome duplication in teleosts [23]. Our results indirectly support a previous study that the V1R family expanded when vertebrates adapted to the terrestrial environment [10].

The most surprising, and arguably the most interesting, results from this study are the lack of V2R-like sequences identifiable from the currently available genomic resources for the sea lamprey. The V2R family is believed to encode receptors for detection of water borne compounds [8,10]. This family is characterized by rapid gene turnover and lineage specific phylogenetic clustering [8], and is most expanded in fish and frogs [10]. We anticipated that this family would be represented and expanded in the lamprey genome. Our search for potential V2R orthologs in the current sea lamprey draft genome, and those of other groups [11,13,14], did not produce an identifiable full or partial V2R candidate, nor were any V2R-like transcripts found in olfactory organ EST library (18,815 transcripts), filtered 454 sequences (373,391 transcripts) from adult and parasite olfactory organ, or in the NCBI Trace database for lamprey (19,177,230 traces). It is also not likely that our search parameters were too stringent, as our queries using V2R sequences returned metabotropic glutamate receptors (2 intact genes, 13 pseudogenes) and calcium sensing receptors (2 intact genes) that are most similar to known V2R receptors. Additionally, searches of urochordate and cephalochordate genomes for potential V2R genes have identified no putative V2R sequences [11,14]. These results from urochordate, cephalochordate, and the current sea lamprey draft genomes, as well as olfactory organ transcriptome data suggest that the V2R gene family arose and diversified in the lineage leading to teleosts after the separation of jawed and jawless vertebrates. This provides evidence regarding the timing of the V2R expansion and its' potential role in the successful radiation of the modern vertebrate lineage.

The limited repertoire of CR genes in the lamprey genome, in both the number of gene families and the restricted degree of expansion in each CR family, accounts for the limited number of odorants that are detected by lamprey. The 59 intact CR genes described in this study likely represent a large portion of the complete CR repertoire of sea lamprey, for two reasons. Extensive and stringent alignment and mapping analysis using lamprey ESTs indicate that the assembled contiguous sequences represent a minimum of 76 percent of the sea lamprey genome. The majority of lamprey ESTs that do not map to the assembly appear to be non-coding RNAs from those same repetitive regions that have confounded efforts to assemble larger scaffolds. Moreover, the CR transcripts detected in olfactory organ cDNA by 454 sequencing analysis all mapped to the draft genome, suggesting that 76% coverage provides a cautious estimate for coverage of CR families. Therefore, the complete sea lamprey CR repertoire is likely much smaller than those of teleost fish and tetrapods. Electrophysiological studies indicate that the sea lamprey can detect all of the classes of odorants that are detected by teleosts [18]; the actual number of individual odorant compounds within each odorant class that are detectable by the sea lamprey, however, is much reduced [18].

The lack of identifiable V2R homologs in the sea lamprey genome provides a perspective on development of CR early in the vertebrate lineage that leads to the dynamic and vagile life histories that characterize modern vertebrates. Similar to cephalochordates and urochordates, all extant agnathan species spend the largest portion of their life cycles, or their entire life, amongst the benthos. The diversification of CR genes in jawed vertebrates, including the advent of the V2R gene family, likely corresponded to an increased ability in ancient vertebrates to locate food and mates while exploiting novel habitats and avoiding potential threats. In retrospect, this was one of several critical early developments in our lineage that lead ultimately to the diversity of modern vertebrates.

## Conclusion

Analysis of available sea lamprey genome data has identified a minimum of 59 intact single-exon chemosensory receptor genes. These GPCRs are homologous to the olfactory receptors, trace amine-associated receptors and V1R pheromone receptors described in higher vertebrates. Expression of seventeen of these receptors has been detected in the olfactory organs of mating- and parasitic-phase lamprey by 454 sequencing and confirmed by RT-PCR. RT-PCR further indicates that more of these CRs are expressed at relatively low levels in the olfactory epithelia. In situ hybridization for representative TAAR and OR genes in olfactory epithelium demonstrates this expression occurs specifically in the olfactory receptor neurons of adult lamprey. These results provide strong support for the majority of these genes functioning in the sensory epithelium of the olfactory system. The presence of these gene families in the sea lamprey genome also suggests that the advent of OR, TAAR and V1R gene families had occurred before the divergence of jawed and jawless vertebrates.

## Methods

### Parasitic and Adult Lamprey

Adult sea lampreys were collected from tributaries to lakes Huron and Michigan by the staff of the U. S. Fish and Wildlife Service (Marquette Biological Station, Marquette, Michigan, USA). Parasitic sea lampreys were captured in Lake Huron attached to fish caught by commercial and recreational anglers and were collected by the staff of U.S. Geological Survey Hammond Bay Biological Station (Millersburg, Michigan). The animals were transported to Michigan State University and held in flow-through tanks supplied with chilled, aerated well water (5°C).

### Tissue Collection

Olfactory epithelium, brain, liver, kidney, gills and gonads were collected for study. Tissues were immediately placed in liquid nitrogen for DNA and RNA preparation. Olfactory organs were dissected to include the accessory olfactory organ that is immediately adjacent and posterior to main olfactory epithelia. Tissues for cryosectioning were embedded in Tissue Tek O.C.T compound (Sakura Finetek, Torrance, CA) after fixation by 4% paraformaldehyde in phosphate-buffered saline (PBS) pH 7.5.

### Genome Assembly

Whole Genome Shotgun assembly of the sea lamprey (*Petromyzon marinus*) genome was undertaken with sequence data generated by the Washington University Genome Sequencing Center (WUGSC). A total of 18,787,613 sequence reads deposited in the NCBI Trace Archive were downloaded; 382,756 were excluded from the assembly for various reasons. The average read length was 660 nucleotides which yields a total input of 12,147,205,620 nucleotides. Genome coverage is between 5.9 and 9.3X depending on the estimated genome size. 93% of the input reads were paired. The assembly was performed with the Arachne WGS assembly program (v3) written at the Broad Institute and was carried out on the Michigan State University High Performance Computing Center (MSU HPCC) Silicon Graphics Altix 3700 BX2. Total running time for the assembly was 494 hours, 38 minutes (20.6 days). Including computer downtime and repeating select steps of the assembly the total time required was approximately 30 days. To estimate the degree of genome coverage a total of 90,955 ESTs were mapped to the draft 2 assembly with the program BLAT [32] using stringent alignment parameters (minimum score = 400, minimum identity = 98%). 61,904 ESTs (70%) mapped to this assembly. These ESTs had previously been clustered using stackPACK [33] which generated 9,649 multisequence consensi from 65,029 ESTs. The other 22,833 remained as singletons. These 32,482 unique sequences were also mapped to the new draft, 19,056 aligned to the assembly; these 19,056 represent 66,738 ESTs (76% of the total).

### Prediction of Chemosensory Receptor Genes

Candidate odorant receptor gene sequences were identified in the WGS Petromyzon marinus Draft Assembly (Draft v.2, 2007-02) and the NCBI Trace database . A flowchart of the gene identification process is provided [see Additional File 6]. TBLASTN  searches were used to identify intact olfactory genes in sea lamprey using known olfactory receptor, trace amine-associated receptor, and pheromone receptor amino acid sequences as queries [Additional File 7]. Contiguous sequences producing alignment hits (E-values < 1 × 10^-10^) were added to a non-redundant list of queries and searching continued in this manner until no new contig hits were found in the assembly. Genes were predicted using GENSCAN [34] and predicted amino acid sequences (>200 aa) were tentatively identified by batch BLASTP searches against the non-redundant (nr) NCBI Protein database. 7-transmembrane domains were confirmed using Phobius  and TMHMM Server v. 2.0 . Gene names are composed of supercontig numbers from the genome draft assembly, followed by gene family designation and initial predicted amino acid length.

### Phylogenetic Analysis

Predicted lamprey CR genes were included for analysis if they possessed start and stop codons, a complete 7-transmembrane domain, and an open reading frame ≥690 nucleotides. CR-like sequences not meeting these criteria were considered pseudogenes. Sequences identified by BLASTP as G-coupled protein receptors that were not potential CR genes, i.e., GABA-receptors, muscarinic acid receptors, and 5-hydroxytryptamine receptors, were excluded from the analysis.

Multiple amino acid and nucleotide data sets were assembled to evaluate the overall topologies of the predicted gene trees in the context of other lineages with relevant estimated divergence times. BLASTN searches of the *Ciona intestinalis v2.0 *and *Branchiostoma floridaev1.0 *draft genomes were performed locally using a representative list of predicted odorant receptors from lamprey in addition to published V1R and V2R from *Danio *and *Rattus*. Putative *Ciona *and *Branchiostoma *pheromone receptor, TAAR and OR sequences were obtained by TBLASTN and TBLASTX searches of the most recent draft genomes available from the Joint Genome Institute  using lamprey CR queries. Searches were also performed of both databases using teleost, sea urchin, and mammalian pheromone receptor sequences as queries. Searches for potential echinoderm CR gene orthologs were carried out using lamprey CRs as BLAST queries against the *Strongylocentrotus purpuratus *GLEAN3 gene predictions available at the Baylor College of Medicine Human Genome Sequencing Center .

Predicted protein sequences of Ca^++^-sensing receptors (CASR) and metabotropic glutamate receptors (MGR) from lamprey were analyzed alongside *Danio*, *Takifugu *and higher vertebrate V2R, CASR and MGR amino acid sequences (n = 86) by neighbor-joining to detect possible clustering of V2R orthologs [Additional File 5] [35,36]. Similar phylogenetic analyses were conducted with lamprey OR and TAAR genes (n = 55, Figure 2), lamprey and vertebrate V1R genes (n = 25, see Additional File 3) and vertebrate OR and TAAR genes with best-hit predicted genes from *Stronglyocentrotus *(n = 204, see Additional File 2). To provide evolutionary context for the predicted CR repertoire in sea lamprey, functional representatives from nine vertebrate olfactory receptor (OR) families representing both Class I and II OR as described by Niimura and Nei [7] were also included in this phylogenetic analyses.

Neighbor-joining trees were constructed using MEGA4 [35,36]. Amino acid sequences were aligned using CLUSTAL V [37], alignment gaps and missing data were eliminated only in pairwise sequence comparisons, and distances were computed using the JTT protein matrix,.

### Preparation of cDNAs for 454 sequencing

cDNA was prepared from pooled olfactory organ RNA from sexually mature female sea lamprey (n = 5) and both male and female parasitic-stage sea lamprey (n = 5, 3 females). Olfactory organ epithelial tissue was flash frozen in liquid nitrogen and stored at -80°C until extraction. Total RNA was extracted using Perfect Pure RNA Tissue Kit (5 Prime, Gaithersburg, MD) according to the manufacturer's protocol. The quality of the extracted RNA was verified by gel electrophoresis and RNA concentration was quantified using a NanoDrop ND-1000 spectrophotometer. 1 ug of total RNA was used as a template for first strand cDNA synthesis using the SMART™ cDNA Synthesis Kit (Clontech Laboratories, Inc.). 13 cycles of LD-PCR was performed to amplify single-strand cDNA using the Advantage^® ^2 PCR Kit (Clontech Laboratories, Inc.) according to manufacturer's instructions. PCR products were purified using QIAquick PCR Purification Kit (Qiagen, Inc) and concentrated on Millipore YM-30 (MWCO 30,000) columns. 5200 ng of cDNA were submitted to the Research Technology Support Facility at Michigan State University for Roche 454 GS 20 sequencing.

### Bioinformatics processing of cDNA reads generated by Roche 454 GS 20

The TIGR SeqClean^1 ^sequence trimming pipeline was used to remove low quality, low complexity, polyA and adapter sequences from the cDNA sequences prior to any analyses. Sequences were aligned to the draft genome assembly (Draft v.2, 2007-02) using BLAT^2 ^[32]and resulting alignments were further filtered using the associated pslSort and pslReps tools. The stringent filtering threshold was set for 95% sequence identity and 90% coverage of the cDNA sequence.

Clustering and consensus assembly of the cDNA sequences was performed using the TIGR Gene Indices clustering tools (TGICL) [38]. Clusters may be generated ab-initio or by using known full or partial cDNA sequences to "seed" the clustering. Two complete 454 runs were performed. After screening for vector, low complexity, low quality sequences, these runs resulted in a total of 373,391 high quality reads with an average read length of 93 nucleotides. This resulted in a total read length of 35,035,388 nucleotides and a total unique length of 10,420,310 nucleotides that were mapped to the assembly. The NJ tree (Figure 2) includes 454 expression data for lamprey OR and TAAR. Table 1 summarizes the available CR expression data.

### RT-PCR Amplification of Predicted Odorant Receptor Genes

To analyze tissue-specific expression of CR genes, primers designed from predicted CR gene sequences were used to perform RT-PCR on RNA samples extracted from different sea lamprey tissues (liver, brain, testis, kidney and gill) of adult and parasitic phases. One μg total RNA from each tissue was reverse-transcribed using oligo (dT) primers. The resulting cDNA was subjected to PCR using the gene-specific primers. A partial β-actin sequence was amplified from the same cDNA template as a positive control. The PCR cycling parameters are as follows: 45 sec. at 94°C, 45 sec. at 52–58°C, 45 sec. at 72°C; 35 cycles; and final step: 10 min. at 72°C. PCR products were visualized on a 1% agarose gel by ethidium bromide staining. Results for 6425.OR330 and 16230.TAAR353 are shown in Figure 3. RT-PCR results, primer sequences and annealing temperatures are shown in Table 1.

### Synthesis of Digoxigenin- labelled cRNA Probes

Digoxigenin labelled antisense RNA probes were generated from sea lamprey OR clones using the Riboprobe In vitro Transcription System (Promega). G_olf _mRNAs are expressed specifically in odorant receptors neurons [39], and because immunoreactive G_olf _are widely distributed in odorant receptor neurons of both larval and adult sea lampreys [40], a positive control probe was generated from a G_olf _cDNA clone kindly provided by Dr. R. Reed (Johns Hopkins University, Baltimore, MD). In brief, 2 μg linearized vector were transcribed in the presence of 700 nmol digoxigenin-11-UTP. The cRNA was collected by ethanol precipitation and resuspended in DEPC water. The sense RNA was prepared by a similar procedure and used as the negative control.

### Tissue Preparation

Olfactory rosettes dissected from adult, parasite and larval lamprey and testis dissected from adult sea lamprey were fixed in 4% paraformaldehyde PBS solution for 3 h. Following cryoprotection in 25% sucrose buffer overnight at room temperature, the tissues were embedded in Tissue Tek O.C.T compound and rapidly frozen in -80°C. Cross sections of 12 μm were cut using Leica1850 cryostat at -25°C, adhered to Superfrost plus microslides (Fisher Scientific; Orangeburg, NY) and stored at -80°C.

### Hybridization

Tissue sections were brought to room temperature, treated with proteinase K (20 μg/ml in PBS) for 5 min and post fixed for 15 min in 4% paraformaldehyde/PBS solution. Sections were rinsed three times for 10 min. each in PBS before a 2 h incubation in prehybridization solution [50% deionized formamide, 1× Denhart's solution, 750 mM sodium chloride, 25 mM ethylenediaminetetraacetic acid (EDTA), 25 mM piperazine-N,N'-bis (2-ethanesulfonic acid; PIPES), 0.25 mg/ml calf thymus DNA, 0.25 mg/ml Poly A acid and 0.2% sodium dodecyl sulfate (SDS)]. Sections were then hybridized with antisense or sense RNA probe in hybridization solution (prehybridization solution containing 5% dextran sulfate) at 60°C for 16–20 h. After hybridization, sections were washed three times for 10 min each in 2×SSC with 0.3% polyoxyethylenesorbitan monolaurate (Tween-20) followed by three washes in 0.2×SSC with 0.3% Tween-20 at 65°C.

### Immunovisualization of Digoxigenin

For detection of digoxigenin-labeled probes, the sections were blocked for 1 h in 4% dry milk, 2% bovine albumin and 0.3% triton. The sections were incubated for 3 h with alkaline phosphatatase-conjugated sheep anti-digoxigenin F_ab _fragments, 1:1000 in blocking solution (Boehringer Mannheim; Indianapolis, IN). The color was developed with incubation in nitroblue tetrazolium chloride and 5-bromo-4-chloro-3 indolyl phosphate substrate (NBT/BCIP, Boehringer Mannheim) for 20–30 min. Sections were mounted in DPX Mountant.

## Abbreviations

CR: chemosensory receptor; TAAR: trace amine-associated receptor; OR: odorant receptor; CASR: calcium-sensing receptor; MGR: metabotropic glutamate receptor; GPCR: G protein-coupled receptor; EST: expressed sequence tag; ORN: olfactory receptor neuron; ISH: in situ hybridization.

## Authors' contributions

SL, CW and WL designed research, SL and KC assembled genome and 454 transcriptome data and performed data mining, HW, SL and YWCD performed RT-PCR and ISH, JT designed the ISH study, ZZ generated Sanger EST libraries, SL and WL wrote the manuscript. All authors read and approved the final manuscript.

## Supplementary Material

Additional file 1 **Nucleic acid and peptide sequences of predicted intact single exon chemosensory receptors from the sea lamprey genome**. Nucleotide and peptide sequences in FASTA format. Click here for file

Additional file 2 **NJ analysis of deuterostome CR genes**. Neighbor-joining analysis including all intact lamprey OR and TAAR genes, representatives of Class I and II ORs from teleosts and tetrapods, and sea lamprey nearest-neighbor GLEAN-predicted chemosensory genes from the genome of the sea urchin *Strongylocentrotus purpuratus*. The urchin chemosensory genes form a single well-supported group whose position suggests a possible independent origin for echinoderm rhodopsin-type amine chemosensory receptors. Click here for file

Additional file 3 **NJ analysis of V1R from lamprey and representative gnathostomes**. Neighbor-Joining analysis of putative lamprey V1R genes including teleosts and tetrapod representative V1Rs. Distances computed using the JTT matrix are included and statistical support in the unrooted tree is presented as a percentage of 1000 bootstraps. Click here for file

Additional file 4 **Lamprey and teleost V1R peptide sequence alignment**. CLUSTALV Alignment of teleost V1R gene with lamprey V1R amino acid sequences. An alignment with a single teleost V1R (AAX10116) is included to emphasize the distant homologies in the V1R genes. Annotations from previous studies that allowed for the identification of putative V1R genes in *Petromyzon *are shown. Sites conserved in mouse V1Rs, N-linked glycosylation sites [22], and those sites differentially conserved in fish and mammal OR and V1r-like genes [7,23] are highlighted. Click here for file

Additional file 5 **NJ analysis of V2R, CASR and MGR from lamprey and representative gnathostomes**. Neighbor-Joining analysis of V2R-, metabotropic glutamate- and calcium-sensing receptor amino acid sequences from sea lamprey and representative gnathostome taxa. Statistical support in the unrooted tree is represented by percentage of 1000 bootstrap replicates with distances computed by the JTT matrix method (MEGA). All positions containing gaps and missing data were eliminated from the dataset. 396 positions were analyzed in the final dataset. Click here for file

Additional file 6 **Identification of CRs expressed in the lamprey olfactory organ**. Strategy used to identify the sea lamprey chemosensory receptor gene repertoire and to survey their expression in olfactory organ cDNA and NCBI EST databases. Click here for file

Additional file 7 **Supplemental Sequences from Niimura and Nei (2006) and Shi and Zhang (2007) used as queries for BLASTN, TBLASTN, PSI-BLAST searches and for the construction of position-specific scoring matrices (PSSMs)**. Complete list of nucleotide and amino acid sequences used to query the sea lamprey draft genome for potential chemosensory receptor genes. Click here for file
