# Supplementary figures and images for: The sea lamprey Petromyzon marinus genome reveals the early origin of several chemosensory receptor families in the vertebrate lineage
Source: BMC Evol Biol. 2009 Jul 31;9:180. doi: 10.1186/1471-2148-9-180 (PMC2728731; doi:10.1186/1471-2148-9-180)

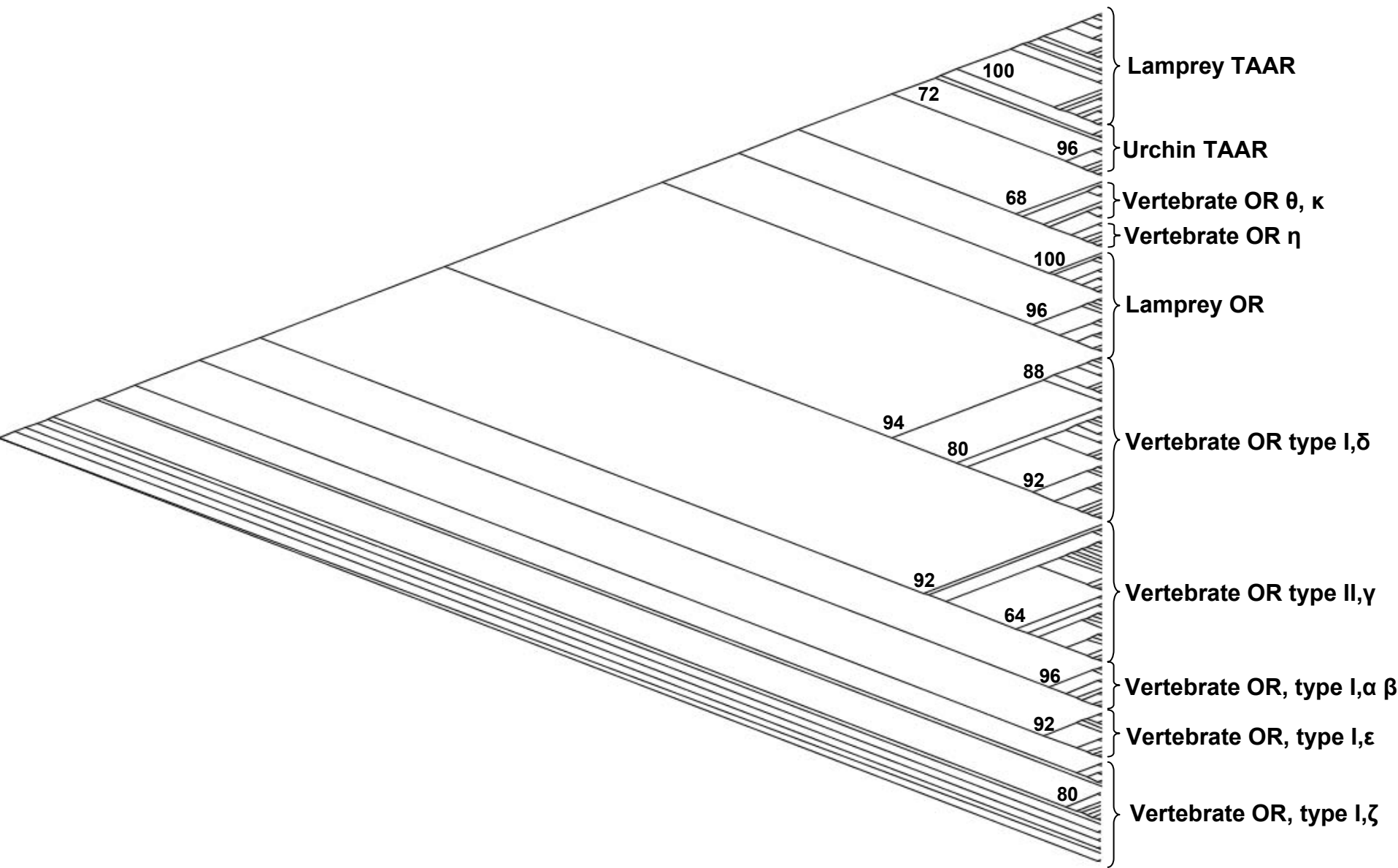

Supplement: Additional file 2 — NJ analysis of deuterostome CR genes. Neighbor-joining analysis including all intact lamprey OR and TAAR genes, representatives of Class I and II ORs from teleosts and tetrapods, and sea lamprey nearest-neighbor GLEAN-predicted chemosensory genes from the genome of the sea urchin Strongylocentrotus purpuratus. The urchin chemosensory genes form a single well-supported group whose position suggests a possible independent origin for echinoderm rhodopsin-type amine chemosensory receptors. [file 1471-2148-9-180-S2.pdf]

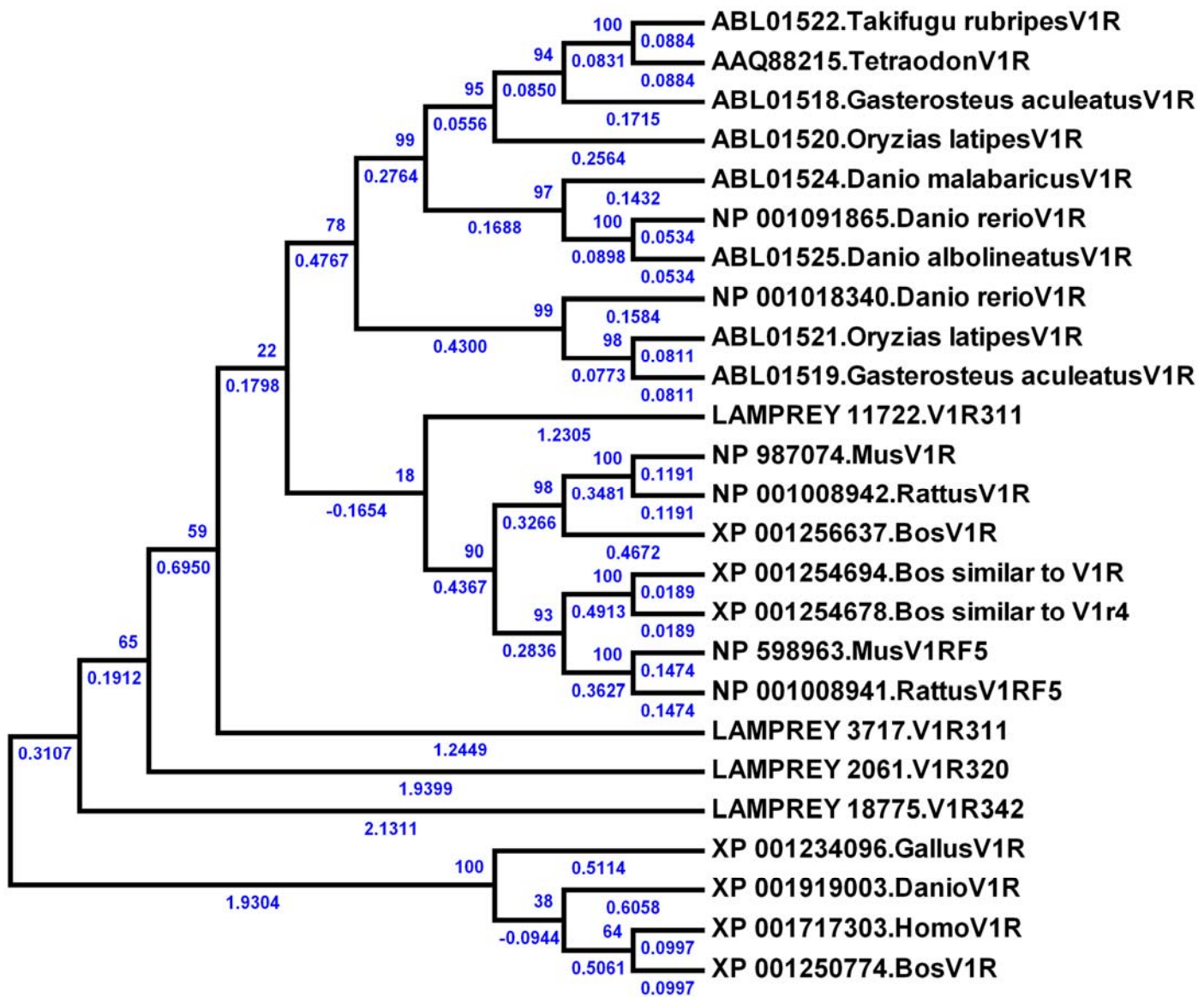

Supplement: Additional file 3 — NJ analysis of V1R from lamprey and representative gnathostomes. Neighbor-Joining analysis of putative lamprey V1R genes including teleosts and tetrapod representative V1Rs. Distances computed using the JTT matrix are included and statistical support in the unrooted tree is presented as a percentage of 1000 bootstraps. [file 1471-2148-9-180-S3.pdf]

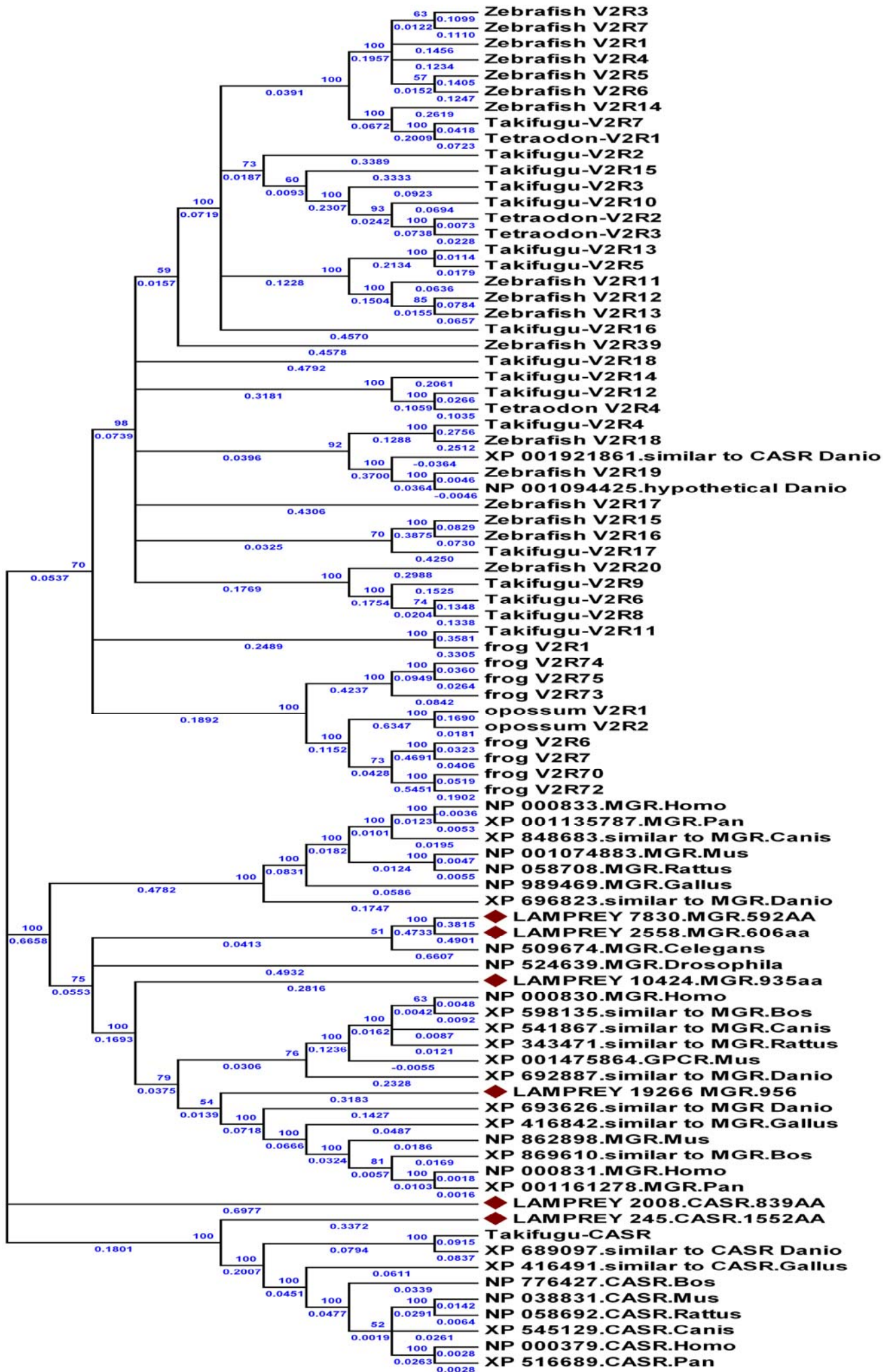

Supplement: Additional file 5 — NJ analysis of V2R, CASR and MGR from lamprey and representative gnathostomes. Neighbor-Joining analysis of V2R-, metabotropic glutamate- and calcium-sensing receptor amino acid sequences from sea lamprey and representative gnathostome taxa. Statistical support in the unrooted tree is represented by percentage of 1000 bootstrap replicates with distances computed by the JTT matrix method (MEGA). All positions containing gaps and missing data were eliminated from the dataset. 396 positions were analyzed in the final dataset. [file 1471-2148-9-180-S5.pdf]

## IN SILICO

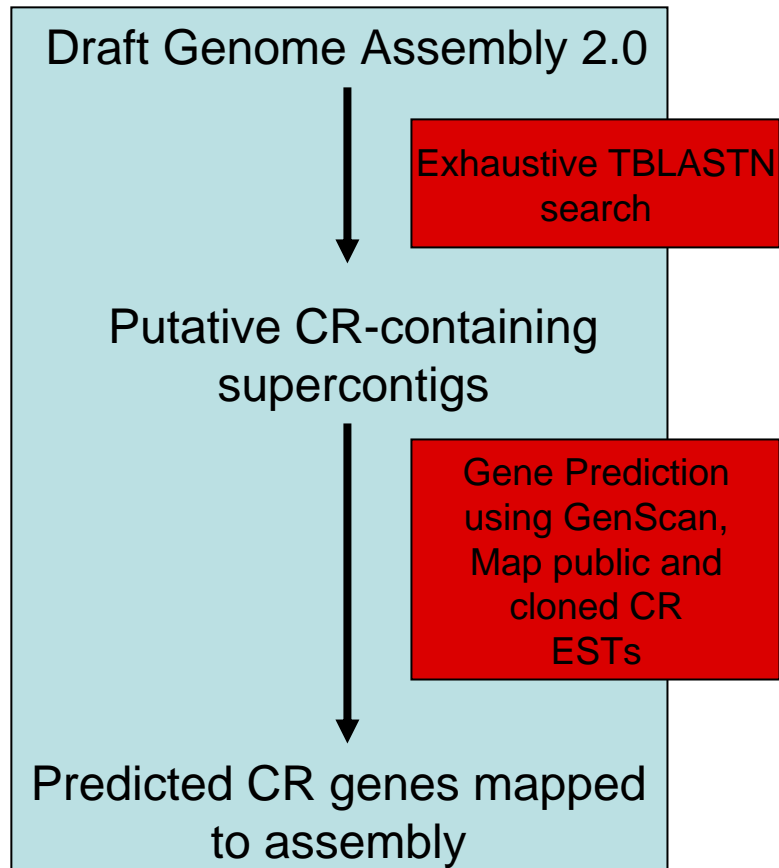

## IN VIVO

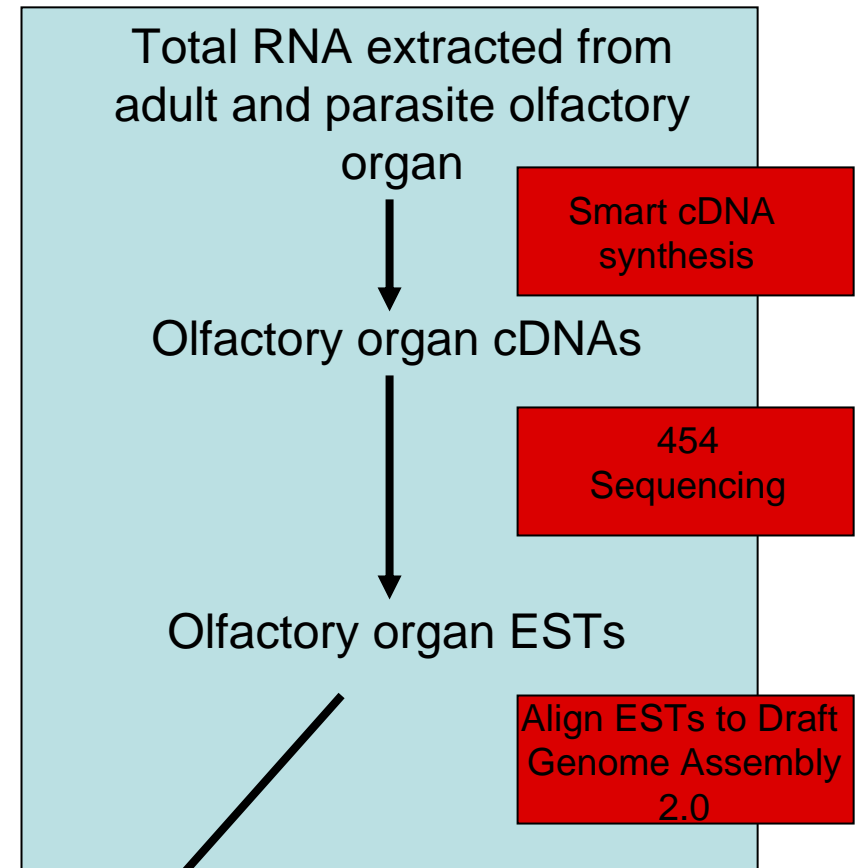

Predicted CR genes expressed in sea lamprey olfactory organ

Supplement: Additional file 6 — Identification of CRs expressed in the lamprey olfactory organ. Strategy used to identify the sea lamprey chemosensory receptor gene repertoire and to survey their expression in olfactory organ cDNA and NCBI EST databases. [file 1471-2148-9-180-S6.pdf]
