# Supplementary material for: The sea lamprey Petromyzon marinus genome reveals the early origin of several chemosensory receptor families in the vertebrate lineage
Source: BMC Evol Biol. 2009 Jul 31;9:180. doi: 10.1186/1471-2148-9-180 (PMC2728731; doi:10.1186/1471-2148-9-180)
Supplement: Additional file 4 — Lamprey and teleost V1R peptide sequence alignment. CLUSTALV Alignment of teleost V1R gene with lamprey V1R amino acid sequences. An alignment with a single teleost V1R (AAX10116) is included to emphasize the distant homologies in the V1R genes. Annotations from previous studies that allowed for the identification of putative V1R genes in Petromyzon are shown. Sites conserved in mouse V1Rs, N-linked glycosylation sites [22], and those sites differentially conserved in fish and mammal OR and V1r-like genes [7,23] are highlighted. [file 1471-2148-9-180-S4.pdf]

Lamprey V1R aligned to V1r pheromone receptor-like AAX10116 [Danio malabaricus]

```
 Score = 75.5 bits (184),  Expect = 6e-12, Method: Compositional matrix adjust.
 Identities = 70/280 (25%), Positives = 135/280 (48%), Gaps = 24/280 (8%)
Query  17    GTIGNTCVLTVFLHIAVKNGELLPSDRSVINMSIVHLLASVFKNILYVFDFFKMH-LLNN  75
             G + NT VL   + HI + + +L P D  + +++    L+  + + +       F M  LL++
Sbjct  1     GILTNTLVLLAYTHIRLTDSQLQPVDAILCHLAFADLMLLLTRCVPQTMTVFGMRNLLDD  60

Query  76    IGCRLLLYSINLFRTLSISFTLLLSLFQFNKLQNSTYPGINVISLKFEKSICLVSVLLWI  135
              GC++++Y+   + RTLS+  T +LS+FQ    +      P ++ +  +  + +    V LW
Sbjct  61    AGCKVVIYTYRIARTLSVCITCMLSVFQAVTVAPEAGPQLSRLKARLPQLVTPTFVALWF  120

Query  136   ISSAIFTPLLFFDDILTLNNSSGLLQFSSCSISPSGGYHLYFGIGTIILVDGFG------  189
             I+ A+      FF    ++    +G +    + ++    G  H+ F        +++G
Sbjct  121   INMAVCIAAPFF----SVAPRNGTVPPFTLNL---GFCHVDFRDNLSYVINGVAVSVRDF  173

Query  190   AF--AIVTMNVLILKILWKHHEMVAARGWVHNANHMATLNAVKIVACLLITYIICWSINI  247
             AF   A++   +   IL +L +H    V   RG    +          A + V   L++ Y + + I+
Sbjct  174   AFVGAMLGSSGYILVLLHRHGRRV--RGIRRSQGSTMETRAARTVEMLVVLYAVFFGIDN  231

Query  248   IIRILLMRFGASLSAIP--MADIASVFSSLYYSFSPFIII  285
             +I I ++     ++  +P   +AD+    FSS Y S SPF+II
Sbjct  232   VIWIYML----TVPQVPPVVADMRVFFSSCYASVSPFLII  267

                                                                           ^    ^
 ^conserved in Mus V1Rs (Pfister and Rodriguez)                   AAX10116 G-ILTN- 5
  conserved in fishes                                           3717.V1R311 G-TIGN- 21
  N-linked glycosylation site                                  11722.V1R311 G-VVVN- 23
  Not conserved in ORs (Saraiva and Korsching)                 18775.V1R342 G-FVGN- 48
  Conserved in ORs (Niimura and Nei)                            2061.V1R320 NLFMTAP 39
  In fish V1R but not mammal V1R (Niimura and Nei)                           .  :

gi|59891918|gb|AAX10116.1|      ------TLVLLAYTHIRLTDSQLQPVDAILCHLAFADLMLLLTRCVPQTM 49
3717.V1R311                     ------TCVLTVFLHIAVKNGELLPSDRSVINMSIVHLLASVFKNILYVF 65
stig_11722.V1R311               ------ALILGVIANTACAYECLPPSDAILVNLAFVNLLISLVRNGPLLF 67
stig_18775.V1R342               ------SLVI-IITGFKIKKS--TPT-ILIMNLAIADFILILTLPLWIVH 88
stig_2061.V1R320                SLCLFLSITVTIFTEVKLREN---SRYILFASLLACDTFFLLCSTIMSTF 86
                                      :  :              .    .  :   . :  :

                                             ^           ^          ^
gi|59891918|gb|AAX10116.1|      TVFGMRNLLDDAGCKVVIYTYRIARTLSVCITCMLSVFQAVTVAPEAGPQ 99
3717.V1R311                     DFFKM-HLLNNIGCRLLLYSINLFRTLSISFTLLLSLFQFNKLQNSTYPG 114
stig_11722.V1R311               WDAGVRMSLSDVGCKLCMGVWVVTRSVSVWTTLTLSMFHYLMVS-RRHTS 116
stig_18775.V1R342               YALDYHWPYGHLMCKIMPFISYVNVTASIYFVTTLSLDRFLLAV--QHPW 136
stig_2061.V1R320                IWLQTNLSLTS--CSVNTMVSTILNQSGVLCVTLMSVERYVAIC---HP- 130
                                             * :       :     .:  .  :*: :          .

gi|59891918|gb|AAX10116.1|      LSRLKARLPQLVTPTFVA-LWFINMAVCIAA--PFFS-VAPRNGTVPPFT 145
3717.V1R311                     INVISLKFEKSICLVSVL-LWIISSAIFTPL--LFFDDILTLNNSSG--L 159
stig_11722.V1R311               VKGKTSALRN-AALTLLC-VWAANVLFSLPA--VVFSGNSAGNFTERLML 162
stig_18775.V1R342               VPKIRT-MNN-TILVCFG-VWLFASLLSIPT--FVFSKALEHNGQVYCYQ 181
stig_2061.V1R320                FHHPRC-FSHRKTLKFLAIIWSVASVFPLACGILVFTSGVTVNNTRT--- 176
                                .      : :       .  :*     .   .    .*       *

                                           ^
gi|59891918|gb|AAX10116.1|      LN------LGFCHVDFRD---NLSYVINGVAVSVRDF-AFVGAMLGSSGY 185
3717.V1R311                     LQ------FSSCSISPSGG-YHLYFGI-GTIILVDGFGAF--AIVTMNVL 199
stig_11722.V1R311               VSGTVRPLLG-CVLHFASAREGLAYTT--ASMIINEIVPIV-LMVTVNIK 208
stig_18775.V1R342               DND----LVDHAAIILAN--FVINFLI---PITIMSF-CYLYIVLKMSRV 221
stig_2061.V1R320                -----------CSTYLTD-----SLMP--NPMAITVLRESP-NVIIFSVC 207
                                           .     .              : :  :       ::  .

                                    ^  ^                            ^          ^
gi|59891918|gb|AAX10116.1|      ILVLLHRHGRRV--RGIRRSQGSTMET-RAARTVEMLVVLYAV--FFGID 230
3717.V1R311                     ILKILWKHHEMVAARGWVHN-ANHMATLNAVKIVACLLITYII--CWSIN 246
stig_11722.V1R311               TLLILHRHRRQLHVASLMPS-RVSTEV-KAAKVIMVVVTIFVI--CWGIH 254
stig_18775.V1R342               SYSANRRAINLIIAIVVVFC-LCWLPY-FLVDIVEGSLILHDVNKYWHVS 269
stig_2061.V1R320                IIALIFTYFNIMMAARKASDDASKAKR-AKQTVILHGLQLFLY--LCSLG 254
                                         . :                        :   :  .       :

gi|59891918|gb|AAX10116.1|      N----VIWIYM-LTV--PQVPPV--VADMRVFFSSCYASVSPFLII---- 267
3717.V1R311                     I----IIRILL-MRFG-ASLSAIP-MADIASVFSSLYYSFSPFIIIFGRS 289
stig_11722.V1R311               -----VFAINY-YNY--RQDDDLEWLISLARFSTAINISMGPLILVVGHG 296
stig_18775.V1R342               HETNKAKRILISLAFSHSCLNPIL-YAFLAKGFRTQFKS--AFILQYFEH 316
stig_2061.V1R320                N---RFFEILFAIIT--TDRNMIT-ILRLCN-YLCLYYS-SRFVVPLIYG 296
                                        *              :      :          *   :::
```
